# Stress‐induced increase of monoclonal antibody production in CHO cells

**DOI:** 10.1002/elsc.202100062

**Published:** 2022-02-24

**Authors:** Jana Schellenberg, Tamanna Nagraik, Ole Jacob Wohlenberg, Sebastian Ruhl, Janina Bahnemann, Thomas Scheper, Dörte Solle

**Affiliations:** ^1^ Institut für Technische Chemie Leibniz Universität Hannover Hannover Germany; ^2^ Field Application Specialist – Cell Culture Technologies Sartorius Stedim Biotech GmbH Göttingen Germany

**Keywords:** cell size, cell‐specific productivity, CHO, mAb, stress

## Abstract

Monoclonal antibodies (mAbs) are of great interest to the biopharmaceutical industry due to their widely used application as human therapeutic and diagnostic agents. As such, mAb require to exhibit human‐like glycolization patterns. Therefore, recombinant Chinese hamster ovary (CHO) cells are the favored production organisms; many relevant biopharmaceuticals are already produced by this cell type. To optimize the mAb yield in CHO DG44 cells a corelation between stress‐induced cell size expansion and increased specific productivity was investigated. CO_2_ and macronutrient supply of the cells during a 12‐day fed‐batch cultivation process were tested as stress factors. Shake flasks (500 mL) and a small‐scale bioreactor system (15 mL) were used for the cultivation experiments and compared in terms of their effect on cell diameter, integral viable cell concentration (IVCC), and cell‐specific productivity. The achieved stress‐induced increase in cell‐specific productivity of up to 94.94.9%–134.4% correlates to a cell diameter shift of up to 7.34 μm. The highest final product titer of 4 g/L was reached by glucose oversupply during the batch phase of the process.

**Abbreviations**
CHO: chinese hamster ovary
FMA: feed media A
FMB: feed media B
IgG_1_
: immunoglobulin G1
IVCC: integral viable cell concentration
mAb: monoclonal antibody
PM: production media
VCC: viable cell concentration

## 1 INTRODUCTION

Nowadays, monoclonal antibodies (mAbs) production in mammalian cell culture is mainly performed with fed‐batch platform processes in large stirred tank reactors [1]. Due to their ability to grow in suspension in a chemically defined medium and the human‐like glycosylation pattern, recombinant Chinese hamster ovary (CHO) cells make up 70% of the used production organisms for therapeutic proteins [2, 3]. Although CHO cell lines have proven their advantages in the biopharmaceutical industry, a remarkable research effort is still directed at understanding and optimizing established cell culture processes [4]. One approach includes studying different modes of operation. For instance, continuous processes such as perfusion have shown to reach viable cell concentrations (VCCs) of up to 200 million cells/mL and product yields of 1.9 g/L*d [5, 6, 7] compared to 26 million cells/mL and a titer of up to 13 g/L in fed‐batch processes [8, 9, 10]. Furthermore, hybrid strategies combining perfusion and fed‐batch operation as well as semiperfusion processes report increased product yield and viable cell density [11, 12]. A second approach is to increase the cell‐specific productivity in the existing benchmark fed‐batch process. Intensified processes and related high cell densities entail challenges regarding the aeration [13], nutrient supply and cell retention, which can be avoided by increasing the individual productivity of already existing cells in a fed‐batch process [14, 15, 16, 17, 18]. As a result, a standardized VCC would yield a higher product titer. Previous studies showed an increase in productivity during the stationary phase and the transition phases into and out of it [19]. Importantly, the methods applied in this second approach should not impair cell growth in its exponential phase to ensure high cell concentrations during the time of highest productivity. Also, as cells supposably increase in diameter depending on certain cell cycle phases [20, 21], detecting high producing cells via size distribution of the average cell diameter seems feasible.

CHO cells do not have a cell wall and, as a consequence, are very susceptible to cell death by shear stress and mechanical stress factors. Metabolic stress factors might have a different impact on the cultivation process performance instead. The intracellular metabolism of mammalian cell lines is relatively unknown despite the intense focus on CHO cells as the preferred host cell line for recombinant protein production [22]. Modern techniques such as metabolic engineering aim to increase product titer, quality, and overall process performance but are limited due to insufficient knowledge on mammalian cells’ metabolism [23]. In terms of nutrient supply, glucose has been recognized as the primary nutrition and energy source for proliferation and cell maintenance. A study by Newsholme et al. suggests further considering the amino acids glutamine and glutamate equivalently vital for cell growth and protein production [24]. While glutamine is widely accepted to be essential for cell proliferation and metabolism maintenance, a direct provision of L‐glutamate can potentially increase productivity by skipping the first step of the used catabolic pathway.

Several studies show a correlation between increased cell size and increased specific productivity (*Q*_P_) induced by hypothermia [25], cell cycle arrest [26], or hyperosmotic stress [27]. A linear correlation of enhanced *Q*_P_ to cell enlargement was found after high methotrexate (MTX) administration using the same transfection method in nine different CHO cell clones [28]. Furthermore, enhancement of cell functions such as transcription, translation, and secretion lead to a directly related increase in cell volume and in *Q*_P_ [29, 30].

**PRACTICAL APPLICATION**

CO_2_ feed and macronutrient supply were investigated regarding their effects on cell growth, cell size, and cell‐specific productivity. Our research reveals a stress related increase in mAb production for pH shifts and CO_2_ supply reduction, glucose overfeeding, and increased glutamate supply as well as the importance of glutamine for proliferation. Together with the discovery of a nonlinear correlation between cell enlargement and increased specific productivity, this marks the first step toward better process understanding and optimization.

This study aims to evluate stress factors as deviations from the standard process that induce an increase in cell‐specific productivity of mAbs to further optimize a fed‐batch process with CHO DG44 cells. Here, CO_2_ feed and macronutrient supply were mainly considered. Additionally, a suspected relation between cell size and increased protein production is confirmed which can be used to detect high producer cells.

## 2 MATERIALS AND METHODS

### 2.1 Cell lines and medium

A CHO DG44 cell line (Sartorius Stedim Cellca GmbH) producing an immunoglobulin G1 (IgG1) mAb was cultivated in chemically defined media and feeds of the Sartorius Stedim Cellca Platform. This includes stock medium for the seed culture (SMD), production media (PM) for the main culture and two different feed media for macronutrients like glucose (feed medium A, FMA) and micronutrients like amino acids (FMA and feed medium B, FMB).

### 2.2 Seed culture

After thawing a cryovial of 30 million cells/mL residues of the freezing media were removed by centrifugation of the cells for 3 min at 190×*g* (Micro Star 17, VWR). The supernatant was decanted, and the cell pellet resuspended in prewarmed SMD and transferred into a 500 mL Erlenmeyer shake flask (Corning) containing 150 mL SMD in total. For five passages of 3–4 days, the shake flasks were incubated (Heracell 240, Thermo Fischer) at 36.8°C, 7.5% pCO_2_, and 85% humidity with a shaking rate of 120 rpm before inoculation of the production culture.

### 2.3 Shake flask studies

Shake flask cultures were inoculated with a seeding density of 0.3 million cells/mL into 150 mL PM using 500 mL baffled shake flasks (Corning) with a vent cap. All triplicate standard cultivations were performed at 36.8°C (±0.5°C), 7.5% pCO_2_, 85% humidity, and 120 rpm in an incubation shaker (Heracell 240, Thermo Fischer). The process lasted 12 days with a 3‐day batch phase in the beginning and daily feeds at the following process days (Supporting Information, Table S1).

### 2.4 Small‐scale bioreactor studies

An Ambr 15 with up to 24 disposable cell culture bioreactor vessels (Sartorius Stedim Biotech GmbH) with a Rushton impeller was used. Independent gassing for each bioreactor of O_2_, CO_2_, and N_2_ enable a fully automated and individual process control of pH and dissolved oxygen. Temperature and stir speed were controlled for each culture station containing up to 12 vessels. Optical DO and pH spots are already implemented into the vessel to measure online. Sampling was performed automatically by a liquid handler and measured offline.

The production culture was inoculated with a starting cell concentration of 0.3 million cells/mL into 12 mL PM. All standard cultivations were performed at an agitation of 1050 rpm and temperature at 36.8°C (±0.5°C) with setpoints for pH at 7.1 (±0.0) and DO at 60% (±20%), respectively. To prevent foaming a 2% solution of Antifoam C Emulsion (30%, Sigma) was added every 12 h. The process lasted 12 days, starting with a 3‐day batch phase in the beginning and daily feeds at the following process days (Supporting Information, Table S1). Due to the low bioreactor volume and relatively high sample volume, samples for the nutrient screening experiments were taken on day 0 and then from day 5 onward.

### 2.5 Offline analytics

Process specific metabolite concentrations including glucose, lactate, glutamine, and glutamate in the cell‐free supernatant were analyzed with the YSI Select 2900D (Xylem) or Cedex Bio Analyzer (Roche). Analysis of the VCC, the viability according to the total cell concentration, and the average cell diameter was performed with a Trypan Blue Assay based Cedex HiRes Cellcounter and Analyzer system (Roche). Trapezoidal integration of the VCC over time results in the integral viable cell concentration (IVCC) which was calculated according to Equation (1) [31].

(1) $$\begin{array}{ccc} \mathrm{IVC}C_{n} & = & \int_{t\;=\;0}^{t}\mathrm{VCC}\left(t\right)dt\approx \sum_{i\;=\;1}^{n}\left(\frac{\mathrm{VC}C_{i}+\mathrm{VC}C_{i-1}}{2}\right)\\ & & \times \;\left(t_{i}-t_{i-1}\right) \end{array}$$

### 2.6 Quantification of IgG_1_

High‐performance liquid chromatography (HPLC) was performed using a VWR Hitachi HPLC System (VWR International) with a Yarra 3 μm SEC 3000 (Phenomenex Inc.) for SEC‐HPLC and a POROSA 20 μm (Thermo Fisher Scientific) for Protein A chromatography. A combination of 0.1 M Na_2_SO_4_ and 0.1 M Na_3_PO_4_ (Sigma) with a pH at 6.6 was used as a buffer for SEC‐HPLC. For a product quantification via Protein A column, a buffer system with a pH of 7.9 containing 0.5 M NaH_2_PO_4_, 0.5 M Na_2_HPO_4_, and 0.5 M NaCl was used for equilibration and a buffer with 0.1 M glycin and 0.5 M NaCl at pH 2 for elution. Before analysis, all samples were diluted to a product concentration of up to 0.5 g/L and filtered through Millex‐GV 0.22 μm syringe filters (Merck) into 0.3 mL HPLC vials (VWR International) using 1 mL syringes (Injekt‐F). Samples of the pH shift experiments were measured by an automated photometric turbidity assay using the Cedex Bio Analyzer.

### 2.7 Calculation of cell‐specific productivity

To compare different bioreactor systems, the daily cell‐specific productivity (*Q*_P_) was calculated according to Equation (2) with the product concentration c and the VCC and then averaged [32].

(2) $$Q_{Pi}=\;\frac{c_{i}-c_{i-1}}{t_{i}-t_{i-1}}\;{\left(\frac{\mathrm{VC}C_{i}+\mathrm{VC}C_{i-1}}{2}\right)}^{-1}$$

Additionally, the product titer at the end of each cultivation was divided by the respective IVCC for comparison of the overall specific productivity. Here, both values will be referred to as cell‐specific productivity.

## 3 RESULTS AND DISCUSSION

**FIGURE 1:**
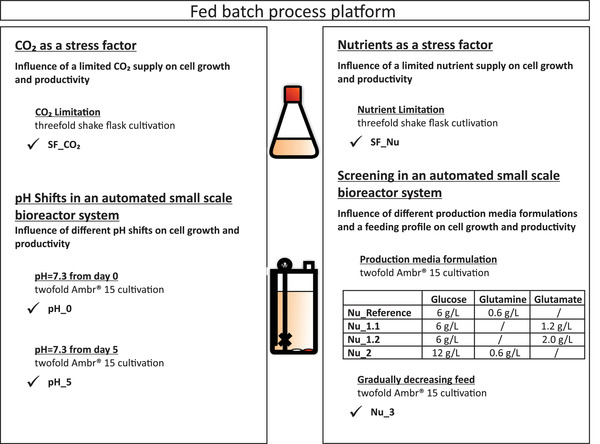
Overview of this work experimental structure

This study investigates the effects of different stress factors on the cell‐specific productivity of a CHO DG44 cell line producing a monoclonal IgG_1_ antibody. Changes of the CO_2_ or pH as well as nutrient supply by different media compositions and feeding strategies were mainly considered as shown in Figure 1.

Both stress factors (CO_2_ and nutrient supply) were investigated at first in shake flasks and afterwards more detailed in the automated Ambr 15 bioreactor system. The CO_2_ supply limitation in shake flasks corresponds to the pH shift in the small‐scale bioreactors. For the nutrient screening experiments, nutrition limitation by a prolonged batch phase in shake flasks as well as different feeding profiles and base media compositions in the Ambr 15 were investigated.

### 3.1 First insights into limitation of CO_2_ and nutrients as stress factors

To establish a reference fed‐batch process (Supporting Information, Table S2) the data of a threefold shake flask cultivation was averaged and used to compare cell growth, cell‐specific productivity as well as cell diameter and metabolites. For the investigation of CO_2_ supply limitation, shake flask cultivations with CO_2_ supply reduction during the last three passages of the seed culture as well as the main culture were performed as shown in Table 1. The used incubator's CO_2_ feed was decreased to simultaneously perform the experiment in threefold.

**TABLE 1:** Process conditions of the shake flask cultivations shown in Figure 2

ID	Process conditions
SF_Reference	Standard process as reference
SF_CO_2_	CO_2_ limitation
SF_Nu	Prolonged batch phase

**FIGURE 2:**
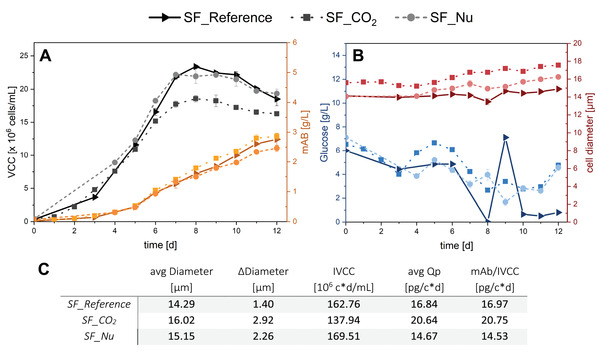
VCC and product concentration (mAB) for SF_CO_2_ with CO_2_ limitation and SF_Nu with nutrient limitations compared to the SF_Reference (A). Glucose concentration and average cell diameter for CO_2_ and nutrient limitation compared to the SF_Reference (B). Calculated values for overall average cell diameter, max. difference in cell diameter, IVCC, cell‐specific productivity *Q*_P_, and specific productivity with regard to product titer and IVCC (C). mAB, monoclonal antibody; IVCC, integral viable cell concentration; VCC, viable cell concentration

By a prolonged batch phase (first feed at day 4 instead of day 3) the second stress factor was also investigated in shake flasks (SF_Nu). With this delay in feeding a limitation of nutrients was introduced.The results for cell growth, product concentration, cell diameter, and glucose concentration of all shake flasks cultivations are shown in Figure 2.

The effects of CO_2_ limitation are clearly visible in reduced growth and increased diameter of the cells. SF_CO_2_ reached a peak VCC of 18.68 mio cells/mL with a significantly lower IVCC compared to the SF_Reference (23.93 mio cells/mL). Although cell growth is reduced, the cell‐specific productivity increased by 22.3–22.6%, resulting in a final product titer of 2.86 g/L which is similar to the SF_Reference of 2.79 g/L.

The opposite can be observed for SF_Nu, where the VCC reached up to 22.46 mio cells/mL with an IVCC comparable to the SF_Reference. Regarding the productivity however, a slight decrease of 12.8–13.4% becomes visible resulting in an mAb titer of 2.46 g/L.

These experiments reveal a CO_2_ stress‐induced enhanced productivity and cell enlargement with lower cell growth. In contrast to this, a negative impact of a nutrition limitation on cell‐specific productivity rather than cell proliferation can be observed. As it is very difficult to measure pCO_2_ in shake flasks and CO_2_ is part of the media buffer system (acid), changes of the pH value were investigated as a possible response to CO_2_ supply reduction. To investigate if direct changes of the easily controlled and monitored pH also result in heightened productivity and cell size, experiments with pH shifts were conducted in the Ambr 15 system.

### 3.2 Investigation of pH shifts as a response to CO_2_ limitation

**FIGURE 3:**
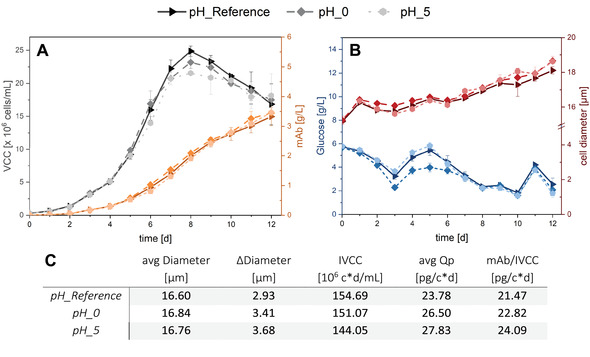
VCC and product concentration (mAb) for pH_0 and pH_5 with pH shifts compared to the pH_Reference (A). Glucose concentration and average cell diameter for pH shifts compared to the pH_Reference (B). Calculated values for overall average cell diameter, max. difference in cell diameter, IVCC, cell‐specific productivity *Q*_P_, and specific productivity with regard to product titer and IVCC (C). mAB, monoclonal antibody; IVCC, integral viable cell concentration; VCC, viable cell concentration

The automated Ambr 15 bioreactor system was used to investigate the effect of a heightened pH from the start of the cultivation and from day 5 onward in twofolds (Table 2). This time was chosen due to the visible beginning of a deviation from the SF_Reference in the preceeding shake flask experiment SF_CO_2_ (Figure 2). Four vessels under standard process conditions were used as reference. The results for cell growth, product concentration, cell diameter, and glucose concentration are shown in Figure 3.

**TABLE 2:** Process conditions of the cultivations in Ambr 15 shown in Figure 3

ID	Process conditions
pH_Reference	Standard process as reference in Ambr 15
pH_0	pH 7.3 from day 0
pH_5	pH 7.3 from day 5

For both cultivations with pH changes a slight deviation from the pH_Reference (pH = 7.1) regarding the cell growth can be observed. The decrease is more evident in pH_5 than in pH_0 with peak VCCs of 21.54 and 23.17 mio cells/mL, respectively (pH_Reference: 24.86 mio cells/mL). As expected, the effect on cell‐specific productivity and cell size is reciprocal. Here, an increase of 6.3–11.4% (pH_0) and up to 17% (pH_5) was reached by a pH shift from 7.1 to 7.3. For pH_5 deviations from the pH_Reference in cell growth and cell size are visible from day 5 up to the end of the cultivation, confirming the pH shift as the cause of changes in cell metabolism. The enlargement of the cells correlates with increased productivity.

The smaller deviations of cell proliferation and mAb production in comparison to SF_CO_2_ suggest a more severe pH shift in the shake flask experiments. Both experiments with the later pH shift (SF_CO_2_ and pH_5) show a higher productivity than the experiment with a pH shift from the beginning (pH_0). This leads to the assumption, that a change during the cultivation generates more cell stress than constant cultivation conditions. Similar effects of a pH shift can be found for the production of an anti‐CD52 mAb in CHO cells [33]. Additionally, lower CO_2_ flow rates were required to maintain a higher pH of 7.3 which conversely confirms that CO_2_ supply reduction leads to pH changes in the culture medium (Supporting Information, Figure S1).

### 3.3 Optimization of feeding profiles and supply of a nitrogen source

During the first insight in shake flasks a nutritient limitation resulted in reduced productivity. Therefore, a nutritient oversupply is investigated as a stress factor. For media screening experiments in the small‐scale bioreactor Ambr 15 system, the results of four vessels under standard process conditions were used as reference. Every other process condition was performed in twofold. First, the formulation of the PM was changed according to Table 3. As a second approach, an oversupply followed by a gradually decreasing feed media volume (FMA and FMB) was tested (Supporting Information, Table S1).

**TABLE 3:** Process conditions of the cultivations in Ambr 15 shown in Figure 4

ID	Process conditions
Nu_Reference	Standard process as reference in Ambr 15
Nu_1.1	No glutamine, but glutamate in base medium (1.2 g/L)
Nu_1.2	No glutamine, but glutamate in base medium (2 g/L)
Nu_2	2 × Glucose concentration in base medium (12 g/L)
Nu_3	Gradually decreasing feed

As high glutamine concentrations support lactat production which has to be avoided, two different increased glutamate concentrations (1.2 and 2 g/L) as replacements for glutamine in the PM are investigated for the first experiments (Nu_1.1/1.2). Here, the effect of glutamate on cell growth and productivity as well as the absence of glutamine during the batch phase was studied.

**FIGURE 4:**
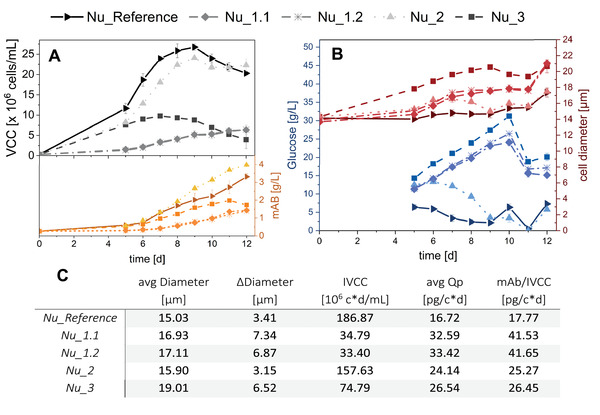
VCC and product concentration (mAb) for Nu_1.1, Nu_1.2, Nu_2, and Nu_3 with different PM formulations and feeding strategies compared to the Reference in (A). Glucose concentration and cell diameter with different PM formulations and feeding strategies compared to the Reference in (B). Calculated values for average cell diameter, max. difference in cell diameter, IVCC, cell‐specific productivity *Q*_P_, and specific productivity with regard to product titer and IVCC are shown in (C). mAB, monoclonal antibody; IVCC, integral viable cell concentration; PM, production media; VCC, viable cell concentration

To investigate the effects of glucose overfeeding during the batch phase the PM was spiked with glucose to contain 12 g/L (Nu_2). Furthermore, a heightened but gradually decreasing feed in Nu_3 achieves a nutrient overfeed in the beginning of the fed‐batch phase. For these experiments the aim is to examine nutrient oversupply during different cell growth phases. The results of all nutrient screening experiments are shown in Figure 4.

For all experiments, a negative impact on cell growth can be observed. In Nu_1.1 and Nu_1.2, glutamate was added to the PM instead of glutamine as a nitrogen supply. Both cultivations show the slowest cell growth and thus the lowest VCCs of all experiments in this work with a maximum of 6.37 mio cells/mL for Nu_1.1 and 6.58 mio cells/mL for Nu_1.2 (Nu_Reference: 26.74 mio cells/mL). This can also be observed in a decrease in IVCC of about 82%. Furthermore, there is no significant difference observable between the two.

For Nu_2, the cells were overfed with glucose (12 g/L) on day 0 of the cultivation which led to a prolonged lag phase in cell growth. A maximum VCC of 24.07 mio cells/mL was achieved on day 9, which is only slightly lower than the Nu_Reference. Therefore, the IVCC shows the smallest decrease of these experiments (15.6%). A nutrient oversupply from day 3 in Nu_3 led to significantly slower cell growth during the exponential growth phase with a comparable VCC to Nu_2 (12 g/L glucose in PM) on day 5. A maximum VCC of only 9.79 mio cells/mL was achieved on day 7 with a decline afterwards. As a result, the IVCC decreased by about 60% in comparison to the reference.

Regarding the cell‐specific productivity, the experiments with glutamate instead of glutamine (Nu_1.1/1.2) show the highest increase of 94.9–133.7% and 99.9–134.4%, respectively, suggesting an importance of glutamate combined with the absence of glutamin during the batch phase for protein production. However, since the cell growth was the lowest, mAb titers of only 1.43 g/L (Nu_1.1) and 1.39 g/L (Nu_1.2) were reached. Here, the relatively high start concentrations and the low end titer affect the integral calculation method of the cell‐specific productivity significantly. Product concentration on day 0 can be set to ∼0 g/L to reduce the difference between mAB/IVCC and avg *Q*_P_ values. For all other experiments, the resulting changes in mAb/IVCC are negligible. The highest product end titer of 3.98 g/L was achieved in Nu_2 (12 g/L glucose in PM) due to the high VCC in combination with an increase of 42.2–44.4% in cell‐specific productivity. For a heightened but gradually decreasing feed in Nu_3 an interesting trend can be observed. Here, the product concentration is comparable to the Nu_Reference until day 6 although the VCC is significantly lower. However, due to a low end‐viability of 72.2% (Supporting information, Tables S8 and S9) degradation of the product by secreted proteases occurred from day 11 at a maximum of 1.98 g/L product. The mAb titer on day 12 was disregarded for the calculation of avg *Q*_P_ and mAb/IVCC which then shows an increase of 48.8–58.7% in comparison to the Nu_Reference.

Due to the accumulating nutrient oversupply in Nu_3 (decreasing feed), glucose concentrations are the highest of all experiments (max. 31.23 g/L), possibly leading to glucose inhibition and further slowing down cell growth. Glutamine and glutamate concentrations are also slightly higher than the Nu_Reference and increase even further toward the end of the cultivation deviating from a near depletion of glutamate in the Nu_Reference (Supporting Information, Table S3). Furthermore, on day 5 the cells are significantly larger compared to the other experiments and continue to increase in size. Additionally, for Nu_1.1 and Nu_1.2, where glutamine was present in the supernatant but not in any media formulation (Supporting Information, Tables S4–S7) the ability of CHO cells to combine glutamate with NH_3_ to form glutamine can be observed [24]. Since the glutamate concentration in the supernatant also increases over time, the supply of the amino acid was higher than the cells uptake. Nu_1.2 (2 g/L glutamate) shows a glutamine maximum on day 7, followed by a fluctuating trend suggesting simultaneous production and consumption of the amino acid. Additionally, glucose concentrations accumulate to 24.11 and 26.45 g/L respectively and a significant increase in cell diameter from day 5 onward can be observed. The trends of glucose concentration and cell size are comparable to Nu_3 (decreasing feed), where a rapid consumption suggests a heightened cell metabolism around day 10. For the experiment Nu_2, deviations from the Nu_Reference can mostly be observed between day 5 and 9 for cell size and all metabolites except for mAb concentration, suggesting that significant changes may have occurred before day 5. Glucose, however, was rapidly consumed from day 6 onward indicating a high metabolism of the cells and supporting the visibly heightened productivity.

These screening experiments show that a replacement of glutamine with glutamate in the base media enhances the productivity but decreases cell growth significantly. An oversupply of glucose on day 0 increases *Q*_P_ and mAb/IVCC while resulting in comparable VCC thus reaching a higher final mAb titer but prolongs the lag phase for about 1 day. A nutrient oversupply during exponential growth phase negatively affects cell growth and cell viability but temporarily increases productivity.

**FIGURE 5:**
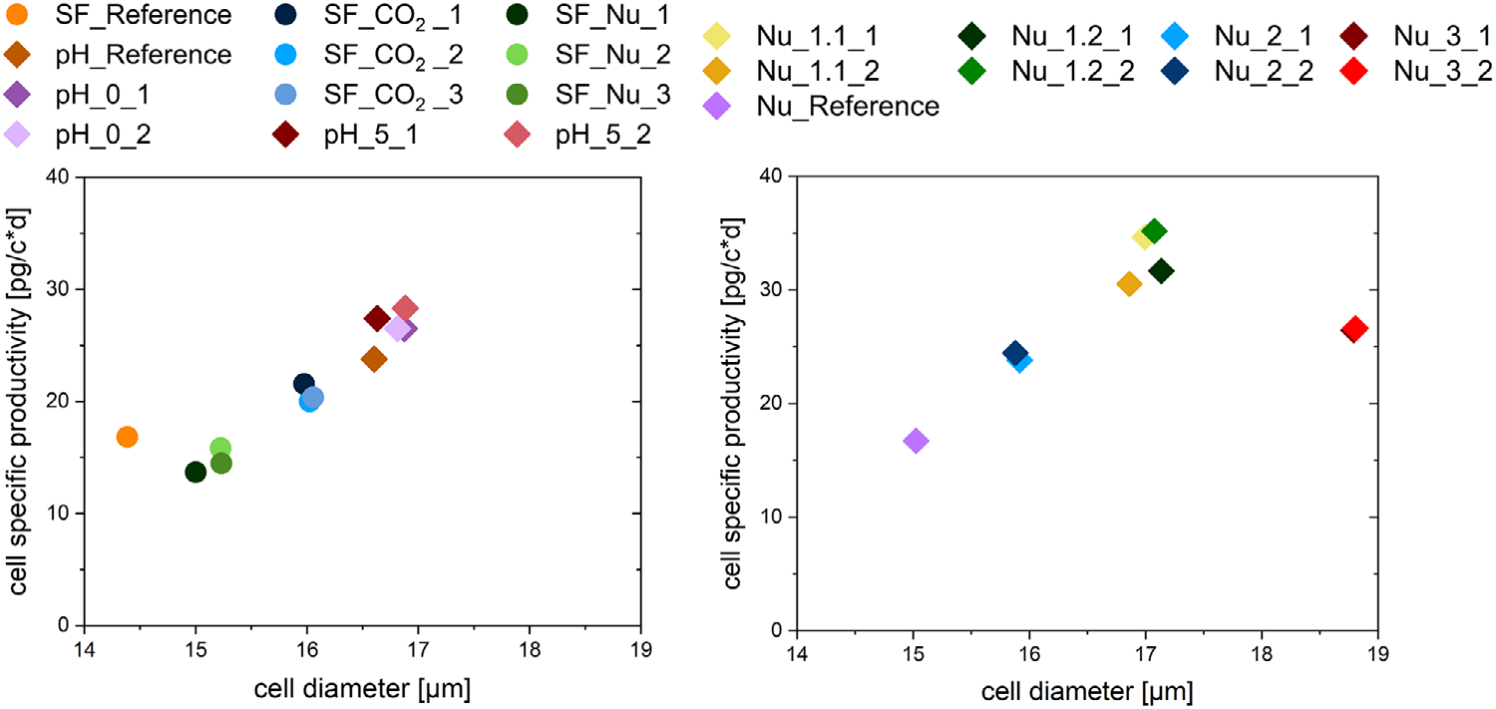
Average cell‐specific productivity (avg *Q*_P_) plotted against avg. cell diameter for threefold shake flask and pH experiments in the Ambr 15 bioreactor system are shown in (A). Avg *Q*_P_ plotted against avg. cell diameter for twofold media screening experiments in the Ambr 15 bioreactor system in (B)

To conclude the presented study, the impact of different process conditions on cell enlargement and the relation to cell‐specific productivity were compared. Figure 5 shows the average cell‐specific productivity (avg *Q*_P_) plotted against the average cell diameter.

In all experiments, the average cell diameter has been increased due to the applied stress factors with high reproducibility for twofold and threefold approaches. Regarding CO_2_ supply reduction, a productivity increase related to cell enlargement can be observed for all shake flasks of SF_CO_2_ in (A). Other experiments with nutrient limitation (SF_Nu) show an increase in cell diameter compared to the reference without heightened protein production suggesting insufficient nutrition of the cells beyond cell maintenance. This implicates the importance of nutrient supply during stressing of the cells to induce amplified cell‐specific productivity. Small shifts to a higher pH value result in a slight increase in cell diameter and productivity with a dependance on the time of the shift (pH_0, pH_5). Additionally, the pH_Reference shows a higher productivity and cell enlargement compared to the SF_Reference. This can partly be caused by the superior process control of the Ambr 15 system. Additionally, a different CHO DG44 cryovial batch was used which can also have an influence.

Media screening experiments regarding nutrient oversupply are shown in Figure 5B, where cells are also generally bigger than in the shake flask experiments. Increased glutamate concentrations in the PM lead to a significant cell enlargement and the highest increase in productivity for twofold of Nu_1.1 and Nu_1.2. Here, no significant difference can be observed between a glutamate concentration of 1.2 g/L (Nu_1.1) and 2 g/L (Nu_1.2). An oversupply of glucose during the batch phase in Nu_2 (12 g/L in PM) also results in a productivity increase with cell enlargement further confirming a correlation between nutrient oversupply and productivity related cell size increase. Yu et al. have shown similar results using highly concentrated feed media [34]. However, since the last experiment Nu_3 shows the biggest size increase of this study, but a comparable avg *Q*_P_ to Nu_2, only a trend of a nonlinear relation can be confirmed.

This study reveals a range of about 15–18.5 μm, where stress‐induced changes in cell size are related to an increased protein production confirming an overall nonlinear correlation between cell enlargement and enhanced *Q*_P_. The findings of cell size related productivity enhancement induced by stress factors are consistent with previous results [20, 26, 27] and can be used as a basis for further process optimization. However, other factors affecting the cell phenotype and diameter such as apoptosis and nutrition supply should also be taken into account since cell enlargement is not always to be equated with high productivity.

## 4 CONCLUDING REMARKS

This study demonstrates the positive impact of different stress factors on the cell‐specific productivity in relation to cell enlargement of a CHO DG44 cell line and forms a basis for further process optimization. pH shifts and CO_2_ supply reduction as well as glucose oversupply induce mAb production. Furthermore, the absence of glutamine combined with added glutamate positively affects cell‐specific productivity while glutamine is necessary for cell growth.

Stressing the cells by CO_2_ limitation and pH shifts generally decreases cell growth but increases productivity and cell diameter. In contrast, a delay in nutrient supply has no significant effect on cell growth but a negative impact on protein production. Additionally, a low end‐viability and an apoptosis related cell enlargement was discovered for a sudden nutrient oversupply at the beginning of the exponential growth phase (Supporting Information, Tables S8 and S9). Here, nutrient accumulation increases cell‐specific productivity while drastically decreasing cell growth, further confirming the connection between cell size and mAb production. The highest increase in cell‐specific productivity (94.9–134.4%), however, was achieved by replacing glutamine with glutamate in the base media formulation simultaneously causing a significant negative impact on cell growth. Finally, the highest cumulative titer in this work (4 g/L) was achieved by a temporary glucose oversupply in the beginning of the cultivation due to increased Q_P_ in addition to high VCC.

Leading forward, the revealed connection between cell enlargement and heightened mAb production allows qualitative detection of induced productivity via cell size using online sensors or flow cytometry. Furthermore, different gassing strategies can be tested regarding a direct influence of pH and oxygen on the cells in a multifactorial DoE. For additional media optimization, a PM formulation with different glutamine concentrations can be tested to optimize the exponential growth phase and achieve higher VCC. Since this study focused on stress from the beginning of the cultivation or during the exponential growth phase, the application during stationary phase is to be investigated. Implementing an additional bolus feed of glucose or glutamate during the stationary phase at high cell concentrations can be applied as a follow up approach in order to achieve higher mAb titers. For insights into the product quality, the bioactivity and posttranslational modifications such as glycosylation and charge variants of the produced mAb should be taken into account.

## CONFLICT OF INTEREST

We confirm that all corresponding authors agree with the submission and publication of this paper and that there is no conflict of interest concerning financial and personal relationships. The manuscript does not contain neither experiments using animals nor human studies. Furthermore, we confirm that the article has not been published previously by any of the authors and is not under consideration for publication elsewhere at the time of submission.

## Supporting information

Supporting Information
Additional supporting information may be found online in the Supporting Information section at the end of the article.

## Data Availability

The data supporting the findings of this study are available on https://doi.org/10.25835/0066265.
