# Supplementary material for: Stress‐induced increase of monoclonal antibody production in CHO cells
Source: Eng Life Sci. 2022 Feb 24;22(5):427–36. doi: 10.1002/elsc.202100062 (PMC9077828; doi:10.1002/elsc.202100062)
Supplement: Supplementary file 1 — Supporting Information Additional supporting information may be found online in the Supporting Information section at the end of the article. [file ELSC-22-427-s001.pdf]

Table S1: Feeding profiles for a standard process and a process with decreasing feed (Nu_3).

	Control feed		Decreasing feed	
	FMA (%)	FMB (%)	FMA (%)	FMB (%)
Day 3	3.98	0.40	10.85	1.09
Day 4	3.81	0.38	9.96	1.00
Day 5	3.66	0.37	9.08	0.91
Day 6	3.52	0.35	8.22	0.82
Day 7	3.36	0.34	7.36	0.74
Day 8	3.22	0.32	6.52	0.65
Day 9	3.08	0.31	5.66	0.57
Day 10	2.96	0.30	4.81	0.48
Day 11	2.85	0.29	3.98	0.40

Table S2: Cultivation Data for the SF_Reference regarding 500mL shake flask scale.

Reference SF	VCC	Viability	Avg Diameter	mAb	Glucose	Lactate	Glutamine	Glutamate
	[x10^6 cells/mL]	[%]	[µm]	[g/L]	[g/L]	[g/L]	[g/L]	[g/L]
Day 0	0.33	99.82	14.13	0.02	6	--	--	--
Day 1	--	--	--	--	--	--	--	--
Day 2	--	--	--	--	--	--	--	--
Day 3	3.65	99.55	13.98	0.14	4.42	1.11	0.05	0.34
Day 4	--	--	--	--	--	--	--	--
Day 5	11.62	99.78	14.14	0.49	4.86	0.49	0.04	0.34
Day 6	16.57	99.72	14.32	0.97	4.86	0.32	0.06	0.37
Day 7	21.83	99.57	14.2	1.27			0.1	0.23
Day 8	23.37	99.36	13.49	1.6	0.02	0.03	0.13	0.04
Day 9	22.44	99.07	14.68	1.84	7.11	0.17	0.17	0.02
Day 10	22.15	97.41	14.44	2.22	0.66	0.4	0.21	0.01
Day 11	20.1	97.73	14.6	2.61	0.52	0.16	0.23	0.04
Day 12	18.51	96.4	14.89	2.76	0.81	0.19	0.27	0.12

Table S3: Cultivation Data for the Nu_Reference regarding the 15mL small scale bioreactor system.

Reference Nu	VCC	Viability	Avg Diameter	mAb	Glucose	Lactate	Glutamine	Glutamate
	[x10^6 cells/mL]	[%]	[µm]	[g/L]	[g/L]	[g/L]	[g/L]	[g/L]
Day 0	0.31	97.80	14.17	0.27	--	--	0.72	0.39
Day 5	11.66	98.40	14.04	0.60	6.40	0.49	0.03	0.43
Day 6	18.67	99.28	14.59	0.73	5.92	0.54	0.07	0.56
Day 7	23.92	99.28	14.81	1.30	3.38	0.44	0.11	0.39
Day 8	25.84	99.33	14.68	1.69	2.39	0.40	0.16	0.15
Day 9	26.74	98.85	14.70	2.02	2.15	0.58	0.19	0.01
Day 10	23.95	98.40	15.40	2.22	6.38	0.32	0.28	0.00
Day 11	21.86	97.08	15.49	2.72	0.49	0.18	0.32	0.00
Day 12	20.30	96.98	17.36	3.32	7.31	0.29	0.43	0.11

Table S4: Cultivation Data for the 15mL bioreactor with 1.2 g/L glutamate and no glutamine in in the PM (Nu_1.1 vessel 1).

Nu_1.1 1	VCC	Viability	Avg Diameter	mAb	Glucose	Lactate	Glutamine	Glutamate
	[x10^6 cells/mL]	[%]	[µm]	[g/L]	[g/L]	[g/L]	[g/L]	[g/L]
Day 0	0.26	92.30	13.89	0.26	--	--	0.04	1.22
Day 5	1.44	95.80	14.71	0.30	11.42	0.15	0.05	1.22
Day 6	1.87	98.90	15.93	0.34	14.16	0.42	0.11	1.61
Day 7	3.09	97.20	16.35	0.41	17.31	0.36	0.12	1.74
Day 8	3.70	96.30	17.45	0.54	19.97	1.02	0.12	1.96
Day 9	4.46	96.90	17.54	0.72	23.78	1.56	0.21	2.05
Day 10	4.53	96.00	18.21	0.81	24.72	1.48	0.02	--
Day 11	5.61	92.50	17.76	1.29	16.30	1.54	0.22	2.03
Day 12	5.55	92.70	21.1	1.38	15.22	1.40	0.35	2.68

Table S5: Cultivation Data for the 15mL bioreactor with 1.2 g/L glutamate and no glutamine in in the PM (Nu_1.1 vessel 2).

Nu_1.1 2	VCC	Viability	Avg Diameter	mAb	Glucose	Lactate	Glutamine	Glutamate
	[x10^6 cells/mL]	[%]	[µm]	[g/L]	[g/L]	[g/L]	[g/L]	[g/L]
Day 0	0.29	88.70	13.49	0.27	--	--	0.03	1.24
Day 5	1.63	95.70	14.59	0.30	11.22	0.16	--	1.39
Day 6	2.45	97.60	15.49	0.35	14.05	0.37	--	1.53
Day 7	3.64	98.80	17.32	0.44	17.33	0.56	0.27	1.67
Day 8	4.52	97.60	16.99	0.57	19.55	1.08	0.24	1.89
Day 9	5.91	99.10	17.58	0.75	22.51	1.46	0.23	2.03
Day 10	6.14	96.70	17.52	1.14	23.49	1.35	0.25	1.90
Day 11	6.73	95.10	17.81	1.43	14.87	1.33	0.33	1.82
Day 12	7.19	95.00	20.96	1.48	15.03	1.30	0.55	2.35

Table S6: Cultivation Data for the 15mL bioreactor with 2 g/L glutamate and no glutamine in in the PM (Nu_1.2 vessel 1).

Nu_1.2 1	VCC	Viability	Avg Diameter	mAb	Glucose	Lactate	Glutamine	Glutamate
	[x10^6 cells/mL]	[%]	[µm]	[g/L]	[g/L]	[g/L]	[g/L]	[g/L]
Day 0	0.29	100.00	13.91	0.25	--	--	0.15	1.92
Day 5	1.39	95.30	15.15	0.30	11.69	0.17	0.03	1.26
Day 6	2.02	98.30	16.03	0.34	14.42	0.38	0.19	1.95
Day 7	3.54	98.10	17.34	0.43	17.88	0.50	0.24	2.14
Day 8	4.24	98.80	17.78	0.60	20.28	1.09	0.30	2.17
Day 9	5.43	98.60	17.91	0.76	23.53	1.60	0.32	2.23
Day 10	5.40	95.30	17.59	0.90	26.36	1.62	0.20	2.31
Day 11	5.90	93.60	17.67	1.06	16.54	1.58	0.30	2.24
Day 12	6.28	94.10	20.84	1.39	16.74	1.56	0.56	2.56

Table S7: Cultivation Data for the 15mL bioreactor with 2 g/L glutamate and no glutamine in in the PM (Nu_1.2 vessel 2).

Nu_1.2 2	VCC	Viability	Avg Diameter	mAb	Glucose	Lactate	Glutamine	Glutamate
	[x10^6 cells/mL]	[%]	[µm]	[g/L]	[g/L]	[g/L]	[g/L]	[g/L]
Day 0	0.28	98.40	13.98	0.25	--	--	0.18	2.00
Day 5	1.28	93.70	15.05	0.26	11.28	0.15	0.23	1.90
Day 6	1.92	97.00	16.03	0.29	14.06	0.33	0.36	2.14
Day 7	2.88	98.40	17.37	0.33	17.55	0.44	0.77	1.68
Day 8	3.86	98.50	17.56	0.65	20.71	0.98	0.46	2.27
Day 9	4.76	97.80	17.44	0.85	24.26	1.55	0.50	2.30
Day 10	4.86	95.90	17.94	0.96	26.55	1.59	0.48	2.26
Day 11	6.15	94.70	17.52	1.35	17.00	1.61	0.49	2.10
Day 12	6.58	95.40	20.79	1.39	17.46	1.63	0.72	2.18

Table S8: Cultivation Data for the 15mL bioreactor with a decreasing feeding profile (Nu_3, vessel 1).

Nu_3 1	VCC	Viability	Avg Diameter	mAb	Glucose	Lactate	Glutamine	Glutamate
	[x10^6 cells/mL]	[%]	[µm]	[g/L]	[g/L]	[g/L]	[g/L]	[g/L]
Day 0	0.28	95.50	14	0.25	--	--	0.74	0.39
Day 5	7.71	98.80	17.78	0.54	14.30	0.74	0.06	1.20
Day 6	8.88	99.20	18.73	0.55	18.35	1.33	0.11	1.51
Day 7	9.98	98.70	19.73	0.98	21.18	1.76	0.16	1.78
Day 8	9.28	98.70	20.16	1.19	24.08	2.08	0.24	1.92
Day 9	8.38	97.20	20.65	1.56	27.81	2.64	0.38	2.36
Day 10	6.74	94.30	19.66	1.76	31.67	2.90	0.47	2.88
Day 11	5.05	88.00	19.62	1.96	19.30	3.89	0.40	2.61
Day 12	2.40	87.70	21.24	1.70	20.79	4.91	0.47	2.99

Table S9: Cultivation Data for the 15mL bioreactor with a decreasing feeding profile (Nu_3, vessel 2).

Nu_3 2	VCC	Viability	Avg Diameter	mAb	Glucose	Lactate	Glutamine	Glutamate
	[x10^6 cells/mL]	[%]	[µm]	[g/L]	[g/L]	[g/L]	[g/L]	[g/L]
Day 0	0.27	94.10	14.69	0.26	--	--	0.68	0.39
Day 5	7.59	97.90	17.9	0.52	14.28	0.80	--	--
Day 6	9.16	98.90	18.86	0.74	18.11	1.35	0.15	1.85
Day 7	9.59	97.80	19.54	0.95	20.98	1.68	--	--
Day 8	9.41	95.50	20.18	1.25	23.76	2.00	0.30	1.94
Day 9	9.22	86.70	20.49	1.60	27.04	2.46	0.59	2.81
Day 10	7.20	74.00	19.65	1.80	30.80	2.70	0.57	2.91
Day 11	5.45	58.30	19.14	1.99	18.37	3.50	0.56	2.81
Day 12	5.53	56.90	20.07	1.75	19.37	4.54	0.63	3.03

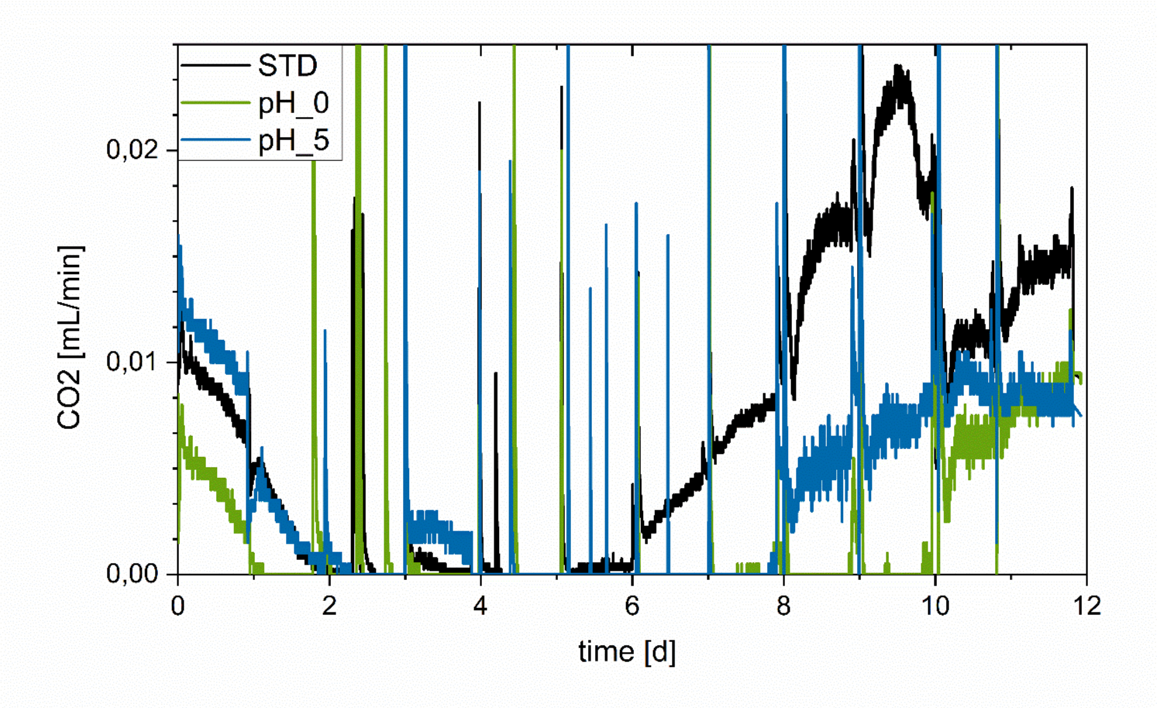

Figure S1: Averaged CO_2 flow rates necessary to maintain the pH set point of different cultivation conditions over time. pH set points were 7.2 for STD (black, fourfold), 7.3 for pH_0 (green, twofold) and 7.2 with a shift to 7.3 from day 5 onwards (blue, twofold).
